# Effects of Nutrition Education Interventions in Team Sport Players. A Systematic Review

**DOI:** 10.3390/nu12123664

**Published:** 2020-11-28

**Authors:** Silvia Sánchez-Díaz, Javier Yanci, Daniel Castillo, Aaron T. Scanlan, Javier Raya-González

**Affiliations:** 1 Faculty of Health Sciences, Universidad Isabel I, 09003 Burgos, Spain; silvia.sanchez2467@ui1.es (S.S.-D.); javier.raya@ui1.es (J.R.-G.); 2 Society, Sports and Physical Exercise Research Group (GIKAFIT), Physical Education and Sport Department, Faculty of Education and Sport, University of the Basque Country, UPV/EHU, 01007 Vitoria-Gasteiz, Spain; javier.yanci@ehu.eus; 3 Human Exercise and Training Laboratory, Central Queensland University, School of Health, Medical and Applied Sciences, Rockhampton 4702, Australia; A.Scanlan@cqu.edu.au

**Keywords:** nutrition knowledge, eating habits, sports performance, body composition, soccer, basketball

## Abstract

Considering nutrition education interventions have been frequently implemented in team sport athletes and have shown promising results, this study aimed to summarize the effects of nutrition education interventions on eating habits, nutrition knowledge, body composition, and physical performance in team sport athletes. A systematic review was conducted using the following databases: PubMed/MEDLINE, Web of Science and SPORTDiscus. A total of 14 studies met the inclusion criteria for the review. The methodological quality of included studies was evaluated, and each study was assessed according to the analyzed variables (i.e., eating habits, nutrition knowledge, body composition, and physical performance). Most studies showed improvements in or maintenance of variables used to indicate eating habits, nutrition knowledge, and body composition. However, limited studies examined the effect of nutrition education interventions on physical performance, with existing studies demonstrating disparate results. These findings suggest implementation of nutrition education interventions in team sport athletes could be an effective strategy to improve their eating habits, nutrition knowledge, and body composition. Due to the heterogeneity across the included studies regarding sport modality, competition level, age, and sex of the athletes investigated, as well as the intervention type adopted (i.e., online or face-to-face), it is difficult to establish optimal nutrition education interventions for each analyzed variable.

## 1. Introduction

Team sports are played around the world [1], accumulating large audiences [2] and generating high economic impact [3]. In recent decades, the application of sport science has become a staple in most team sports to improve the health and performance of athletes [4]. In this sense, sport science research has grown with most studies focusing on three predominant topic areas including: training and game load monitoring through physical, physiological, and perceptual variables [5]; optimization of athletes’ anthropometric characteristics and physical fitness [6]; and evaluation of injury prevention and rehabilitation programs [7]. However, recent research has highlighted the relevance of invisible strategies (i.e., those other than training plans) in the pursuit of optimizing athlete health and performance [8]. Nutrition is considered a key invisible strategy, benefiting performance during competition and facilitating recovery [9].

Most team sport studies focusing on athlete nutrition have reported on the health- and performance-related effects of diets [10,11,12] and supplementation [13,14,15]. However, some promising data have emerged regarding the efficacy of nutrition education interventions in team sports [16]. Nutrition education interventions are specific programs designed to assist target populations in modifying their eating habits and/or enhancing their nutrition knowledge [17]. These outcomes are particularly relevant for team sport athletes given increasing nutrition knowledge can yield substantial positive changes in eating habits in team sport athletes [16]. In turn, improved eating habits can enhance performance in team sport athletes [18]. Therefore, nutrition education interventions appear to be a key strategy to optimize team sport athletes’ performance. Most investigations examining the effectiveness of nutrition education interventions have recruited athletes from individual sports, showing beneficial results in compliance with Mediterranean diet quality index [19] and nutrition knowledge, perceived susceptibility to the Female Athlete Triad, and self-efficacy constructs in specific contexts for each athlete [20]. However, research exploring the application of nutrition education interventions in team sports is more limited than in individual sports, since teams are made up of 15–22 players who have different nutritional needs [21]. Nevertheless nutrition education interventions are being increasingly applied among team sport athletes since interventions can be implemented using a wide range of modalities, including personal interviews [22], group activities [23], comics [24], interactive workshops [23], or technological platforms [25]. However, the effectiveness of each specific modality in nutrition education interventions needs to be analyzed to identify the most appropriate approach relevant to the specific context applied.

Since nutrition education interventions in team sports can benefit athlete nutrition knowledge and dietary behaviors, and in turn performance [26], collating the existing literature on this topic is essential to understand the efficacy of different interventions in improving eating habits in team sport athletes. In this regard, a previous systematic review [18] analyzed the influence of nutrition education interventions on eating habits, finding most studies (13 out of 16 studies) reported positive changes in dietary behavior post-intervention across athletes competing in twenty-five different individual and team sports. Furthermore, meta-analytic evidence [16] showed most nutrition education interventions (85.7%) significantly increased nutrition knowledge in athletes competing in individual and team sports following different nutrition education interventions with face-to-face strategies the most effective intervention modality (28 out of 36 studies). Due to the strong relationships that dietary behaviors and nutrition knowledge hold with body composition and physical performance [9,27,28], many studies have examined the effects of nutrition education interventions on body composition [22,29,30,31,32,33] and performance indicators [32,34,35]. Regarding body composition, Nascimento et al. [31] applied nutrition counselling consisting of four consultations separated by 45 to 60 days in adult and adolescent team and individual sport athletes, observing increased lean body mass in both groups after the intervention (pre-intervention = 48.0 ± 1.2 kg vs. post-intervention = 49.2 ± 1.6 kg; *p* < 0.05). Regarding physical performance, Rossi et al. [32] reported improved change-of-direction speed (change = −0.15 ± 0.13 s; *p* < 0.001) and vertical jump height (change = 6.6 ± 9.4 cm; *p* < 0.001) in collegiate baseball players following a nutrition education intervention involving 90-min information sessions focused on food preparation, nutrients, and healthy eating habits across 12 weeks. Although multiple systematic reviews have explore the effectiveness of education interventions on dietary behaviors and nutrition knowledge [16,18], no systematic reviews have focused explicitly on team sport athletes in this area nor examined the effectiveness of nutrition education interventions on performance indicators in any athlete groups.

Despite the strong interest in conducting nutrition education interventions in team sports, there is currently no scientific consensus for the most effective intervention to apply in practice. Therefore, a systematic review of the literature is essential to generate robust conclusions regarding the effects of different nutrition education interventions in team sport athletes and facilitate their application to practice in specific contexts. Consequently, the aim of this systematic review was to analyze the effects of nutrition education interventions on eating habits, nutrition knowledge, body composition, and physical performance in team sport athletes.

## 2. Materials and Methods

This review was carried out following the recommendations and criteria established in the Preferred Reporting Items for Systematic Reviews and Meta-analysis (PRISMA) statement guidelines [36].

### 2.1. Search Strategy

To identify potential studies, a systematic search was performed in the following databases: PubMed/MEDLINE, Web of Science (including all Web of Science Core Collection: Citation Indexes) and SPORTDiscus. The search syntax included the following keywords with relevant Boolean operators inserted: (“education intervention” OR “nutritional intervention” OR “nutrition education”) AND (“nutrition knowledge” OR “health knowledge” OR “food knowledge” OR “diet knowledge” OR “dietary behavior” OR “dietary behaviour” OR “dietary intake” OR “dietary assessment” OR “food habit” OR “body composition” OR “weight management” OR “performance” OR “physical performance” OR “athletic performance”) AND (“team sport” OR “athlete”). A year restriction was applied for this search (i.e., studies published between 1980 and 2020). Furthermore, the reference lists of included studies were searched, and studies that cited the included studies were located using Google Scholar and checked for their relevance. Two authors (S.S-D. and D.C.) independently screened the title and abstract of each study. Full-text versions of studies that met the inclusion criteria were then screened. Discrepancies regarding inclusion of studies between authors were decided by consensus with a third author (J.R-G.). The search was performed on 21 November 2020.

### 2.2. Inclusion Criteria

Studies meeting the following criteria were included in our review: (1) sample in experimental group was composed solely of team sport athletes; (2) nutrition intervention implemented using educational strategies; (3) instrument(s) used produced quantitative scores pre- and post-intervention; (4) intervention impact was assessed on any of the following outcomes: eating habits, nutrition knowledge, body composition, and/or physical performance indicators; and (5) a full-text version of the study was published in a peer-reviewed journal. In addition, studies that were not written in English, as well as conference abstracts, letters to the editor, errata, narrative reviews, systematic reviews, meta-analyses, or invited commentaries were excluded from our review.

### 2.3. Study Coding and Data Extraction

The following data were extracted from the included studies: (a) authors, year of publication, and study design; (b) sample characteristics (including sample size, sex, age, country, sport modality, and competition level); (c) tool/questionnaire/parameter/test used for each analyzed variable; (d) intervention procedure (modality, frequency, and duration); and (e) major findings (i.e., positive/negative/unchanged effects on eating habits, nutrition knowledge, body composition, and/or physical performance).

### 2.4. Methodological Quality Assessment

To assess the methodological quality of the included studies, a modified version of the Downs and Black checklist was used [37]. In this regard, items 8, 13, and 17 were removed, and the tool was complemented with two additional items (i.e., 9 and 10) from the Academy of Nutrition and Dietetics (AND) quality criteria checklist [38] for greatest relevance to nutrition intervention studies [16]. In the checklist, each question is answered with “yes” if the criteria are satisfied or “no” if the criteria are not satisfied. The answer “yes” was awarded one point for all items (and the answer “no” given zero points) except items 5 and 18, which were scored with a maximum of two points. This approach allowed reviewers to differentiate between studies providing two or less validity or reliability measures for the chosen nutrition knowledge instrument and studies that used three or more validity or reliability measures for the chosen nutrition knowledge instrument. Additionally, for studies using single-arm designs in our review, items 14, 15, 21, 22, 23, and 24 were not deemed relevant, so the maximum score for studies using this study design was 22 points with the following scoring criteria adopted: ≤11 points: poor quality; 12–15 points: fair quality; 16–19 points: good quality; 20–22 points: excellent quality). For studies using double-arm designs, the maximum score was 28 points with the following scoring criteria adopted: ≤14 points: poor quality; 15–19 points: fair quality; 20–25 points: good quality; 26–28 points: excellent quality) [16,39].

Data extraction and methodological quality assessment were performed by two authors (S.S-D. and J.R-G.) independently, and a third author was consulted (D.D.) to solve any discrepancies via consensus. 

### 2.5. Search Results

**Figure 1:**
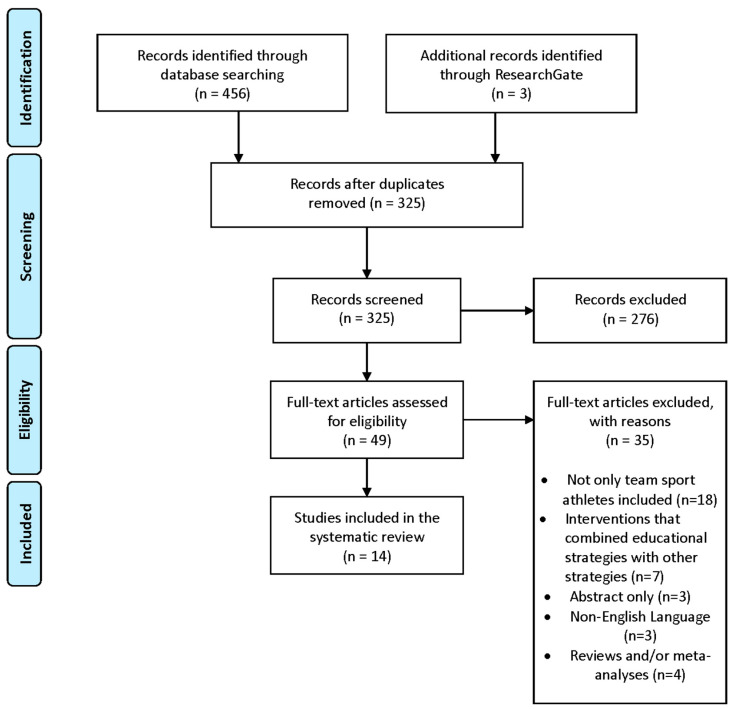
Flow diagram of the study retrieval process.

Figure 1 shows the study retrieval process performed for our review. A total of 456 studies were identified in the initial search, while three additional studies were identified through other sources (i.e., ResearchGate). One hundred and thirty-one study duplicates were eliminated, and 325 studies were screened. Furthermore, 276 studies were excluded based on their titles and/or abstracts. Full-text versions of the remaining 49 studies were assessed for eligibility, with 14 studies meeting the inclusion criteria and being retained in our systematic review [22,23,24,25,26,29,30,32,33,34,35,40,41,42].

## 3. Results

### 3.1. Descriptive Characteristics of the Studies

Table 1, Table 2, Table 3 and Table 4 summarize the characteristics of the 14 included studies according to the outcome variables analyzed. In this regard, 10 studies analyzed the impact of nutrition education interventions on eating habits [22,23,24,25,26,29,30,32,33,40], nine studies analyzed nutrition knowledge [22,23,24,25,26,32,40,41,42], five studies analyzed body composition [22,29,30,32,33], and three studies analyzed physical performance [32,34,35] in team sport athletes. Regarding the experimental design, seven studies used single-arm designs [22,30,32,34,40,41,42] and seven studies used double-arm designs [23,24,25,26,29,33,35]. Moreover, quasi-experimental pre-post and repeated measures designs were used. Included studies examined a total of 683 athletes with five studies examining only male athletes [24,26,30,41,43], six studies examining only female athletes [22,25,29,32,33,40], and three studies examining male and female athletes combined [23,34,35]. Four studies examined volleyball athletes [22,29,33,40], three studies examined soccer athletes [23,24,25], two studies examined field and ice hockey athletes [41,42], one study examined handball athletes [30], one study examined basketball athletes [32], and three studies examined athletes from different team sports [26,34,35]. Finally, interventions between ten days and two seasons were applied, including from 2 to 12 educational sessions and using several delivery modalities (i.e., face-to-face, game, technological platform, workshop, comic book, and sport nutrition lessons).

### 3.2. Methodological Quality Assessment

Table 5 and Table 6 show the methodologic quality of the included studies. In single-arm studies, quality scores ranged from 6 to 16 points, with an average of 11.7 ± 3.4 points, while double-arm studies ranged from 13 to 21 points, with an average of 17.4 ± 2.8 points. Regarding quality assessment of each study, four studies were categorized as poor quality, seven studies were categorized as fair quality, and three studies were categorized as good quality.

## 4. Discussion

The aim of our systematic review was to analyze the effects of nutrition education interventions on eating habits, nutrition knowledge, body composition, and physical performance in team sport athletes. The main results showed that implementation of nutrition education interventions consistently induced positive changes in eating habits and nutrition knowledge, as well as maintained or ameliorated body composition. Meanwhile the disparity of results across a limited number of studies exploring the effectiveness of education nutrition interventions implies further research is needed on this topic to draw definitive conclusions. These findings suggest the health and performance of team sport athletes from various backgrounds could benefit from nutrition education interventions as a complementary strategy to training routines.

### 4.1. Effects of Nutrition Education Interventions on Eating Habits

Balanced nutritional intakes combined with regular physical activity is advisable for optimal growth and health [44,45,46]. Additionally, balanced nutritional intakes are essential to ensure suitable nutrients are available to carry out essential systemic functions in the body, while nutrient deficiencies could influence growth and development as well as negatively affect physical performance in young athletes, and promote greater injury risk in all athletes [47]. Nutrient deficiencies can contribute to the development of certain health problems such as diabetes, obesity, sarcopenia, and osteoporosis [48,49]. In this regard, to improve eating habits by means of appropriate macronutrient and micronutrient intakes is convenient to optimize athlete performance during team sport competition, which could be assisted through nutrition education interventions. Our systematic review collated findings from 13 studies exploring the effects of nutrition education interventions on eating habits in team sport athletes, with ten studies showing total energy, carbohydrate, protein, and fat intakes and eating habits such as daily intake of fruits and vegetables as well as reporting trying to eat for performance were ameliorated in team sport athletes [22,25,26,29,30,31,32,33,40]. Additionally, nutrition education interventions provide team sport athletes with knowledge regarding the nutritional properties of food, which could lead to consumption of varied and balanced diets that provide essential nutrients to enhance performance and recovery. It should also be noted that some major limitations were apparent in one study observing nutrition education interventions to yield no changes in eating habits. In this sense, Patton-Lopez et al. [23] noted athletes in the control group declined the consumption of lunch (consuming lunch on less than five days per week) after the intervention, while athletes in the nutrition education intervention group did not change their consumption (consuming lunch on at least five days per week). NEI athletes were three times more likely to report trying to eat for performance, revealing that these athletes were motivated to learn and improve their diet behaviors and benefit from team-based nutrition interventions. Consequently, our review presents strong evidence demonstrating the effectiveness of using nutrition education interventions to improve eating habits in a diverse range of team sport athletes.

### 4.2. Effects of Nutrition Education Interventions on Nutrition Knowledge

Given greater nutrition knowledge is associated with better eating habits and physical conditioning status [50,51,52], it is relevant for nutrition specialists to ascertain whether nutrition education interventions enhance nutrition knowledge in team sport athletes. In turn, enhanced nutrition knowledge in team sport athletes may promote adoption of a more balanced diet, which could potentially optimize physical preparedness for competition [53]. In this sense, most studies (eight out of nine studies) in our systematic review demonstrated improvements in nutrition knowledge following nutrition education interventions in team sport athletes. Consequently, it appears that team sport athletes develop an understanding of key nutritional concepts following nutrition education interventions to make informed decisions regarding eating habits suited to their training and competition requirements. Contrarily, the study showing no changes in nutrition knowledge following a nutrition education intervention included the shortest intervention period among included studies (i.e., 2 weeks), where 33 Canadian ice hockey athletes completed a sport nutrition workbook in a single-arm design [41]. Consequently, future studies should investigate whether longer nutrition education interventions using various modalities improve nutrition knowledge in ice hockey athletes. Considering the effectiveness of nutrition education interventions on nutrition knowledge across most studies, team sport coaching staff should prescribe additional educational strategies to optimize athlete understanding of nutrition to promote eating behaviors that optimize health and performance across the season.

### 4.3. Effects of Nutrition Education Interventions on Body Composition

Possessing optimal body composition is necessary to perform at a high standard during competition in team sports [28]. Given improved body composition can assist team sport athletes in obtaining better physical conditioning and, consequently, reduce injury risk [47,54], understanding whether nutrition education interventions can enhance body composition in team sport athletes is of interest to coaching staff, athletes, and nutrition specialists alike. All five studies examining the effects of nutrition education interventions on body composition in team sport athletes in our systematic review reported improvements in or maintenance of body composition following the intervention. Three studies showed fat mass to decrease [22,32,33] following nutrition education interventions lasting between 3 and 8 months. In contrast, two studies showed no variations in body composition variables (body mass, body mass index, fat mass, body fat percentage, and sum of skinfolds) following implementation of nutrition education interventions [29,30]. The lack of change in body composition detected in these studies may be attributed to the team sport athletes examined presenting with desired body composition variables at the time of baseline testing given athletes can be required to maintain appropriate physical condition across different seasonal phases to adequately cope with training and competition demands.

### 4.4. Effects of Nutrition Education Interventions on Physical Performance

Team sport athletes should undergo physical conditioning that enables them to meet the physical demands of competition [55] and mitigate the fatigue-related injury risk derived from poor conditions [56]. In this sense, a primary goal of team sport coaching staff is to optimize the physical preparedness of athletes for competition which could be ameliorated with appropriate nutritional intake. Thus, including nutrition education strategies during daily routines could be effective in assisting coaches and athletes in achieving this goal. Our systematic review only retrieved three studies analyzing the effects of nutrition education interventions on physical performance in team sport athletes, with disparity in the reported results due to variations in the characteristics of the athletes examined. In this sense, while some nutrition education strategies improved performance during specific fitness tests (i.e., 600-m running time), no change in performance was observed in other tests (i.e., vertical jump and strength tests). For example, Kavouras et al. [35] observed young volleyball and basketball players to improve 600-m running speed following a hydration intervention plan for 10 days, but no improvements were reported in 30-m linear sprint speed, vertical jump height, and skill performance. Similarly, another study assessing the efficacy of an online sports nutrition curriculum across 8 weeks showed improvements in change-of-direction speed and vertical jump height, with no variations in upper-body strength in male and female team sport athletes [34], while another study demonstrated improvements in change-of-direction speed and vertical jump height in female basketball athletes receiving face-to-face nutrition education for 12 weeks and controls [32]. Considering the mixed results regarding changes in physical performance following nutrition education interventions, further research is needed to build the evidence base for team sport athletes specific to sport modality, competition level, age, and sex to uncover the most appropriate nutrition education intervention to optimize physical performance in different contexts.

### 4.5. Limitations

This study contains some important limitations that should be considered when interpreting the reported effects of nutrition education interventions on eating habits, nutrition knowledge, body composition, and physical performance in team sport athletes. Firstly, it is difficult to establish a definitive consensus regarding specific nutrition education strategies that are most effective at enhancing eating habits, nutrition knowledge, body composition, and physical performance considering the wide range of athletes examined across studies encompassing different sports, ages, competition levels, and cultures. For these reasons, a meta-analysis was not permissible to undertake. Secondly, the small sample of athletes recruited in some studies should be considered when interpreting findings indicating the effectiveness of nutrition education strategies in team sport athletes across the literature. Thirdly, studies included in our review only analyzed physical performance using physical fitness testing, which does not represent actual in-game performance during team sport competition. Consequently, future studies are encouraged examining the effects of nutrition education interventions on in-game performance variables to provide evidence with greater ecological validity on this topic. Fourthly, 11 out of the 14 studies in our review had poor to fair methodological quality suggesting the methodological approaches of studies need to improve on this topic moving forward. Fifthly, the inclusion of athletes from different sports in the control group in some double-arm studies [25,26] should be considered when interpreting the associated results given the variations in physical attributes and nutritional needs that may exist between athletes from different sports [57]. Finally, body composition was measured with different approaches possessing varied validity and reliability across studies including anthropometry, air displacement plethysmography, and dual-energy x-ray absorptiometry, which should be considered when interpreting the findings presented. Future research should expand on the available body composition variables and explore the effects of nutrition education interventions on other variables such as bone mineral content.

## 5. Conclusions

Nutrition education interventions are an effective strategy to improve or maintain eating habits, nutrition knowledge, and body composition in team sport athletes when applied in addition to regular training routines. However, the effects of nutrition education interventions on physical performance in team sport athletes are not clear given the limited studies and mixed findings on this topic. Additionally, due to the heterogeneity across studies included in our review regarding the characteristics of the athletes examined (i.e., sport modality, competition level, age, and sex) and intervention strategies adopted (i.e., modality, frequency, and duration) it is difficult to establish optimal nutrition education interventions to adopt in specific contexts based on the existing literature.

## Figures and Tables

**Table 1:** Characteristics and major findings of the studies analyzing the effect of nutrition education interventions on eating habits in team sport athletes.

Authors	Year	Sample Size (*n*)	Age (M ± SD Years)	Country	Sport	Competition Level	Tool	Intervention (Modality, Frequency and Duration)	Major Findings
Abood et al. [25]	2004	NEI (15 females) CG (15 females)	NEI (19.6 ± 1.0) CG (19.4 ± 1.2)	United States	NEI Soccer CG Swimming	College division I	3-day food record	Nutrition education intervention NEI (online, 8 sessions of 1 h, 8 weeks) CG (no treatment)	NEI athletes experienced a significant increase in self-efficacy compared to CG. CG recorded a decrease in carbohydrate intake (percentage of total calories) and fiber. There was a significant overall difference in the number of positive dietary changes favoring the NEI.
Anderson et al. [29]	2010	Feedback (8 females) Baseline (8 females)	Feedback (20.1 ± 0.5) Baseline (19.3 ± 0.5)	United States	Volleyball	NCAA division II	3-day food record	Feedback regarding dietary intake Feedback (F2F at beginning, peak during, and following each season for two full seasons). Baseline (no treatment)	No changes in total carbohydrate, protein, and fat intake between baseline and feedback across the two seasons; however, greater protein intake was recorded after feedback at beginning of the season.
Daniel et al. [40]	2016	10 females	17.2 ± 0.9	Brazil	Volleyball	Junior	Quesitonnaire of Associação Brasileira de Empresas de Pesquisa	Interdisciplinary Food, Nutrition and Health Education Program (F2F, 8 sessions, 8 months)	Athletes made dietary changes during the year increasing their daily intake of fruits and vegetables and decreasing high-energy, low-nutrient food intake.
Elias et al. [26]	2018	NEI (52 males) CG (53 males)	NEI (18.7 ± 0.9) CG (23.3 ± 3.8)	Malaysia	NEI Field hockey and soccer CG Cricket and rugby union	National	KAP questionnaire	Sport education intervention activities NEI (F2F and online, 7 × 1.5-h sessions, 9 weeks) CG (no treatment)	NEI athletes showed higher mean total energy intake than CG following the education intervention. NEI showed greater increases in total carbohydrate, protein, and fat intake than CG following the education intervention.
Molina-López et al. [30]	2013	14 males	22.9 ± 2.7	Spain	Handball	Professional	3-day food record	Nutrition education program (F2F, 3 phases, 5 months)	Significant increase in total energy and macronutrient intakes following the intervention.
Patton-Lopez et al. [23]	2018	NEI (153 males and females) CG (64 males and females)	NEI (14.9 ± 0.9) CG (14.9 ± 0.9)	United States	Soccer	High school	SNK questionnaire	WAVE education program NEI (F2F, 7 sport nutrition lessons and 3 team-building workshops, 1–1.5 h, 2 years) CG (no treatment)	NEI were three times more likely to report trying to eat for performance. Consumption of lunch (≥ 5-days per week) did not change in NEI, but significantly declined in the CG.
Rossi et al. [32]	2017	15 females	19.3 ± 1.0	United States	Baseball	College division I	3-day food record	Nutrition education intervention (F2F, every 3 weeks, 12 weeks)	NEI athletes significantly increased total energy intake, total carbohydrate intake, and total protein intake following the intervention.
Valliant et al. [22]	2012	NEI 11 females CG 11 females	NEI 19.8 ± 1.0 CG 19.5 ± 1.0	United States	Volleyball	College division 1	3-day food record	Nutrition education NEI (F2F, 4 individual sessions, 4 months) CG (no treatment)	NEI athletes significantly increased total energy, carbohydrate, and protein intake following the intervention.
Wenzel et al. [33]	2012	NEI 10 females CG 29 females	NEI 19.8 ± NR CG 19.8 ± NR	United States	Volleyball	College division 1	3-day food record	Nutrition counselling NEI (F2F, once a month, 4 months) CG (no treatment)	Athletes significantly increased total energy, carbohydrate, and protein intake following the intervention.
Zeng et al. [24]	2020	NEI 15 males CG 15 males	NEI 16.7 ± 1.8 CG 16.8 ± 1.7	China	Soccer	Youth top	3-day food record	Nutrition education intervention NEI (F2F and comic book, once a week during 30 min, 4 weeks) CG (comic book)	No changes were observed in total carbohydrate, protein, and fat intake in NEI and CG following the intervention.

*n*: number; M: mean; SD: standard deviation; NEI: nutrition education intervention; CG: control group; F2F: face to face; KAP: nutrition knowledge, attitude and practice; SNK: sport nutrition knowledge; NCAA: National Collegiate Athletic Association.

**Table 2:** Characteristics and major findings of the studies analyzing the effect of nutrition education interventions on nutrition knowledge in team sport athletes.

Authors	Year	Sample Size (*n*)	Age (M ± SD Years)	Country	Sport	Competition Level	Questionnaire	Intervention (Modality, Frequency, and Duration)	Major Findings
Abood et al. [25]	2004	NEI (15 females) CG (15 females)	NEI (19.6 ± 1.0) CG (19.4 ± 1.2)	United States	NEI Soccer CG Swimming	College division I	Flesch-Kincaid Grade Level Index	Nutrition education intervention NEI (online, 8 × 1-h sessions, 8 weeks) CG (no treatment)	NEI athletes experienced a significant increase in nutrition knowledge and self-efficacy compared to CG.
Daniel et al. [40]	2016	10 females	17.2 ± 0.9	Brazil	Volleyball	Junior	KAP questionnaire	Interdisciplinary Food, Nutrition and Health Education Program (F2F, 8 sessions, 8 months)	NE athletes improved their nutrition knowledge from 57.0 ± 9.9% at baseline to 63.0 ± 11.8% following the intervention.
Elias et al. [26]	2018	NEI (52 males) CG (53 males)	NEI (18.7 ± 0.9) CG (23.3 ± 3.8)	Malaysia	NEI Field hockey and soccer. CG Cricket and rugby union	National	Jürgensen questionnaire	Sport education intervention activities NEI (F2F and online, 7 × 1.5-h sessions, 9 weeks) CG (no treatment)	NEI athletes showed significant increases (−6.21 ± 2.95 points) in nutrition knowledge after the intervention. Significant decrease (−2.15 ± 1.45 points) in nutrition knowledge after the intervention in the CG.
Patton-Lopez et al. [23]	2018	NEI (153 males and females) CG (64 males and females)	NEI (14.9 ± 0.9) CG (14.9 ± 0.9)	United States	Soccer	High school	SNK questionnaire	WAVE education program NEI (F2F, 7 sport nutrition lessons and 3 team building workshops, 1–1.5 h, 2 years) CG (no treatment)	NEI athletes significantly increased their nutrition knowledge scores from 5.16 ± 1.80 points at baseline to 6.09 ± 1.59 points after the 1-year intervention, while there were no changes in the CG.
Reading et al. [41]	1999	33 males	14.3 ± 3.6	Canada	Ice hockey	Youth	Modified version of SNAC questionnaire	Sport Nutrition for the Athletes of Canada (SNAC) (F2F, online and lectures, 4 × 1-h sessions, 2 weeks)	No changes in nutrition knowledge following the intervention.
Rossi et al. [32]	2017	15 females	19.3 ± 1.0	United States	Baseball	College division 1	Reilly and Maughan sports nutrition questionnaire	Nutrition education intervention (F2F, 3 times per week, 12 weeks)	NEI athletes significantly improved their nutrition knowledge from 54.7 ± 14.3% at baseline to 70.0 ± 9.4% following the intervention.
Simpson et al. [42]	2017	17 males	19.0 ± 0.7	New Zealand	Hockey	International and national	Zin et al. questionnaire of nutritional knowledge	Nutrition education intervention (online, once a week, 6 weeks)	NEI athletes significantly improved their nutrition knowledge from 56.7 ± 11.4% at baseline to 61.1 ± 11.5% following the intervention.
Valliant et al. [22]	2012	NEI 11 females CG 11 females	NEI 19.8 ± 1.0 CG 19.5 ± 1.0	United States	Volleyball	College division 1	Reilly and Maughan sports nutrition questionnaire	Nutrition education intervention NEI (F2F, 4 individual sessions, 4 months) CG (no treatment)	NEI athletes significantly increased their nutrition knowledge scores from 24.7 ± 5.9 points at baseline to 31.5 ± 6.1 points following intervention.
Zeng et al. [24]	2020	NEI 15 males CG 15 males	NEI 16.7 ± 1.8 CG 16.8 ± 1.7	China	Soccer	Youth top	KAP questionnaire	Nutrition education intervention NEI (F2F and comic book, once a week during 30 min, 4 weeks) CG (comic book)	NEI improved general and sports nutrition knowledges following the intervention.

*n*: number; M: mean; SD: standard deviation; NEI: sport nutrition education intervention; CG: control group; F2F: face to face; KAP: nutrition knowledge, attitude and practice; SNK: sport nutrition knowledge.

**Table 3:** Characteristics and major findings of the studies analyzing the effect of nutrition education interventions on body composition in team sport athletes.

Authors	Year	Sample Size (*n*)	Age (Mean ± SD)	Country	Sport	Competition Level	Parameter	Intervention (Modality, Frequency, and Duration)	Major Findings
Anderson et al. [29]	2010	Feedback (8 females) Baseline (8 females)	Feedback (20.1 ± 0.5) Baseline (19.3 ± 0.5)	United States	Volleyball	NCAA division II	Body mass Body fat (%)	Feedback regarding dietary intakes Feedback (F2F, at beginning, peak during, and following each season for two full seasons) Baseline (no treatment)	No changes in body mass and body fat percentage at beginning, peak, and following the season between the first season (no information given) and the second season (feedback).
Molina-López et al. [30]	2013	14 males	22.9 ± 2.7	Spain	Handball	Professional	Body mass Body mass index Body fat (%)	Nutrition education program (F2F, 3 phases, 5 months)	NEI athletes showed no changes in body mass index and body fat percentage following the intervention.
Rossi et al. [32]	2017	NEI (15 females) CG (15 females)	NEI (19.3 ± 1.0) CG (19.8 ± 1.4)	United States	Baseball	College division I	Body mass Body fat (%) Fat free mass Fat mass	Nutrition education intervention NEI (F2F, every 3 weeks, 12 weeks) CG (no treatment)	NEI athletes significantly decreased body fat percentage (−1.4 ± 2.2%) following the intervention compared to the CG (0.5 ± 2.6%). NEI athletes significantly decreased fat mass (−1.0 ± 2.0%) following the intervention compared to the CG (0.6 ± 1.4%).
Valliant et al. [22]	2012	NEI 11 females CG 11 females	NEI 19.8 ± 1.0 CG 19.5 ± 1.0	United States	Volleyball	College division 1	Fat free mass (%) Fat mass (%)	Nutrition education NEI (F2F, 4 individual sessions, 4 months) CG (no treatment)	NEI athletes and CG significantly decreased body fat percentage (~2%) and significantly increased fat-free mass percentage (~2%).
Wenzel et al. [33]	2012	NEI 10 females CG 29 females	NEI 19.8 ± NR CG 19.8 ± NR	United States	Volleyball	College division 1	Body mass Body fat (%)	Nutrition counselling NEI (F2F, once a month, 4 months) CG (no treatment)	NEI athletes significantly decreased their body fat percentage (~2%), while CG did not change.

*n*: number; M: mean; SD: standard deviation; NEI: sport nutrition education intervention; CG: control group; F2F: face to face education; F2F: face to face; NCAA: National Collegiate Athletic Association.

**Table 4:** Characteristics and major findings of the studies analyzing the effect of nutrition education interventions on physical performance in team sport athletes.

Authors	Year	Sample Size (*n*)	Age (M ± SD Years)	Country	Sport	Competition Level	Test	Intervention (Modality, Frequency and Duration)	Major Findings
Kavouras et al. [35]	2012	NEI (61 males and females) CG (31 males and females)	NEI (14.0 ± 4.8) CG (13.2 ± 2.3)	Greece	Volleyball and basketball	Youth	600-m run 30-m sprint Vertical jump Skill test	Hydration intervention plan NEI (F2F, 3 times per day, 10 days) CG (no treatment)	NEI athletes significantly decreased 600-m running time (−12.7 ± 1.5%) following the intervention.
Rossi et al. [32]	2017	NEI (15 females) CG (15 females)	NEI (19.3 ± 1.0) CG (19.8 ± 1.4)	United States	Baseball	College division I	5–10–5 shuttle run Vertical jump Broad jump Squat 1RM	Nutrition education intervention NEI (F2F, every 3 weeks, 12 weeks) CG (no treatment)	NEI athletes and CG significantly decreased 5-10-5 shuttle run time (−0.06 to −0.15 s), and significantly increased vertical jump height (5.1–6.6 cm) broad jump distance (9.9–12.0 cm), and 1 RM squat strength (20.0–27.7 kg). No significant (*p* > 0.05) interactions between NEI and CG for each performance variable.
Shoemaker et al. [34]	2019	Male (18) Female (25)	Male (16.6 ± 1.1) Female (16.1 ± 1.0)	United States	School-or club-sponsored sports	NR	Vertical jump Broad jump Agility test Push-up force	Sports nutrition curriculum (online, 7 sessions, 8 weeks)	Athletes increased vertical jump peak power (~13 W·kg^−1^), broad jump distance (~7 cm), and push-up force (~27 N) following the intervention.

*n*: number; M: mean; SD: standard deviation; NEI: sport nutrition education intervention; CG: control group; F2F: face to face education; RM: repetition maximum; NR: not reported.

**Table 5:** Methodologic quality of the included single-arm studies.

Study	1	2	3	4	5	6	7	8	9	10	11	12	13	14	15	16	17	18	19	20	21	22	23	24	25	26	Score (/22)
Daniel et al. (2016) [40]	1	1	0	1	0	1	1	1	0	0	0	0			0	0	0	0	0					0	0	0	6
Molina-López (2013) [30]	1	1	1	1	1	1	1	1	0	0	0	0			1	1	0	1	1					0	1	0	12
Reading et al. (1999) [41]	1	1	1	1	1	1	1	1	1	0	1	1			1	1	1	1	0					0	1	0	16
Rossi et al. (2017) [32]	1	0	1	1	1	1	1	1	1	0	1	1			1	1	0	1	0					0	1	0	14
Shoemaker et al. (2019) [34]	1	1	1	1	1	1	0	1	1	0	0	1			1	1	0	1	1					0	1	0	14
Simpson et al. (2017) [42]	1	1	0	1	0	1	1	0	1	0	0	0			1	1	0	0	0					0	1	0	9
Valliant et al. (2012) [22]	1	1	1	0	0	1	1	1	1	1	0	0			1	1	0	0	0					0	1	0	11

1. Hypothesis stated (/1); 2. Outcome described (/1); 3. Characteristics described (/1); 4. Interventions described (/1); 5. Confounders described (/2); 6. Main findings described (/1); 7. Random variability described for main outcomes (/1); 8. Characteristics of athletes lost to follow-up reported (/1); 9. Actual probability reported (/1); 10. Biases and limitations considered (/1); 11. Representative of population (/1); 12. Participating subjects representative (/1); 13. Attempt to blind subjects (/1); 14. Attempt to blind main outcomes (/1); 15. Data dredging reported (/1); 16. Statistics used appropriate (/1); 17. Compliance with intervention reliable (/1); 18. Measurement tool accurate (/2); 19. Funding reported (/1); 20. Groups recruited from same population (/1); 21. Recruited over same time (/1); 22. Randomization (/1); 23. Intervention assignment concealed (/1); 24. Adjustment for confounding (/1); 25. Athletes lost to follow-up accounted for (/1); 26. Sufficient power (/1).

**Table 6:** Methodologic quality of the included double-arm studies.

Study	1	2	3	4	5	6	7	8	9	10	11	12	13	14	15	16	17	18	19	20	21	22	23	24	25	26	Score (/28)
Abood et al. (2004) [25]	1	1	1	1	2	0	0	1	0	1	0	0	0	0	1	1	1	2	0	1	1	1	0	1	1	1	19
Anderson (2010) [29]	1	1	1	1	1	1	1	0	0	0	0	0	0	0	1	1	0	1	1	1	1	0	0	0	0	0	13
Elias et al. (2018) [26]	1	1	1	1	1	1	1	1	1	1	0	0	0	0	1	1	1	2	0	1	1	1	0	0	1	1	20
Kavouras et al. (2012) [35]	1	1	1	1	1	1	1	1	0	1	0	0	0	0	1	1	1	2	0	1	1	0	0	0	1	0	17
Patton-Lopez et al. (2018) [23]	1	1	1	1	1	1	1	1	1	1	0	0	0	0	1	1	0	1	1	1	1	0	0	0	0	1	17
Wenzel et al. (2012) [33]	1	1	1	1	1	1	1	0	0	1	0	1	0	0	1	1	1	2	1	0	0	0	0	0	0	0	15
Zeng et al. (2020) [24]	1	1	1	1	1	1	1	1	1	1	0	1	0	0	1	1	1	2	1	1	1	1	0	0	1	0	21

1. Hypothesis stated (/1); 2. Outcome described (/1); 3. Characteristics described (/1); 4. Interventions described (/1); 5. Confounders described (/2); 6. Main findings described (/1); 7. Random variability described for main outcomes (/1); 8. Characteristics of athletes lost to follow-up reported (/1); 9. Actual probability reported (/1); 10. Biases and limitations considered (/1); 11. Representative of population (/1); 12. Participating subjects representative (/1); 13. Attempt to blind subjects (/1); 14. Attempt to blind main outcomes (/1); 15. Data dredging reported (/1); 16. Statistics used appropriate (/1); 17. Compliance with intervention reliable (/1); 18. Measurement tool accurate (/2); 19. Funding reported (/1); 20. Groups recruited from same population (/1); 21. Recruited over same time (/1); 22. Randomization (/1); 23. Intervention assignment concealed (/1); 24. Adjustment for confounding (/1); 25. Athletes lost to follow-up accounted for (/1); 26. Sufficient power (/1).
